# Reproducibility of image-based computational models of intracranial aneurysm: a comparison between 3D rotational angiography, CT angiography and MR angiography

**DOI:** 10.1186/s12938-016-0163-4

**Published:** 2016-05-06

**Authors:** Yuan Ren, Guo-Zhong Chen, Zhen Liu, Yan Cai, Guang-Ming Lu, Zhi-Yong Li

**Affiliations:** State Key Laboratory of Bioelectronics, Southeast University, Nanjing, 210096 P.R. China; School of Biological Science and Medical Engineering, Southeast University, Nanjing, 210096 P.R. China; Department of Medical Imaging, Jinling Hospital, Medical School of Nanjing University, Nanjing, 210002 P.R. China; School of Chemistry, Physics and Mechanical Engineering, Queensland University of Technology, Brisbane, QLD 4001 Australia

**Keywords:** Reproducibility, Aneurysm, CFD, Angiography, CTA, DSA, MRA

## Abstract

**Background:**

Reconstruction of patient-specific biomechanical model of intracranial aneurysm has been based on different imaging modalities. However, different imaging techniques may influence the model geometry and the computational fluid dynamics (CFD) simulation. The aim of this study is to evaluate the differences of the morphological and hemodynamic parameters in the computational models reconstructed from computed tomography angiography (CTA), magnetic resonance angiography (MRA) and 3D rotational angiography (3DRA).

**Methods:**

Ten patients with cerebral aneurysms were enrolled in the study. MRA, CTA and 3DRA were performed on all patients. For each patient, three patient-specific models were reconstructed respectively based on the three sets of imaging data of the patient. CFD simulations were performed on each model. Model geometry and hemodynamic parameters were compared between the three models.

**Results:**

In terms of morphological parameters, by comparing CTA based models (CM) and 3DRA based models (DM) which were treated as the “standard models”, the aspect ratio had the minimum difference (Δ = 8.3 ± 1.72 %, P = 0.953) and the surface distance was 0.25 ± 0.07 mm. Meanwhile, by comparing MRA based models (MM) and DM, the size had the minimum difference (Δ = 6.6 ± 1.85 %, P = 0.683) and the surface distance was 0.36 ± 0.1 mm. In respect of hemodynamic parameters, all three models showed a similar distribution: low average WSS at the sack, high OSI at the body and high average WSSG at the neck. However, there was a large variation in the average WSS (Δ = 34 ± 5.13 % for CM, Δ = 40.6 ± 9.21 % for MM).

**Conclusion:**

CTA and MRA have no significant differences in reproducing intracranial aneurysm geometry. The CFD results suggests there might be some significant differences in hemodynamic parameters between the three imaging-based models and this needs to be considered when interpreting the CFD results of different imaging-based models. If we only need to study the main flow patterns, three types of image-based model might be all suitable for patient-specific computational modeling studies.

## Background

Intracranial aneurysms are common neurovascular disease and the most serious consequences are rupture leading to subarachnoid hemorrhage [1]. Hemodynamics may play an important role in the process of aneurysm formation, progression and rupture [2–5]. It has been shown that the size of intracranial aneurysms and other morphological parameters are related to their risk of rupture [6]. Moreover, the change of the hemodynamic parameters, such as wall shear stress (WSS) or wall shear stress gradient (WSSG),may influence the behavior of endothelial cells and smooth muscle cells [7], resulting in flow-mediated vasodilatation and vascular remodeling.

With the development of CT angiography (CTA), magnetic resonance angiography (MRA) and 3D rotational angiography (3DRA) techniques, the image-based patient-specific analysis of the hemodynamics of intracranial aneurysm is becoming important for diagnosis and treatment plan. Image-based analysis of intracranial aneurysms can help with the assessment of the potential risk of aneurysm rupture [8, 9] and provide guidance for treatment choice [10, 11]. Compared with other image technologies, 3DRA can depict considerably more small additional angiographic aneurysms [12, 13]. In addition, the reconstructed 3DRA images can show only the enhanced vascular lumina which allow observing any desired region without hindering over projecting bony structures [14]. Therefore, the diagnosis and measurement of aneurysms can be performed more accurately by using 3DRA technology. However, because 3DRA is invasive and expensive, most clinical imaging for cerebral aneurysm prefer to CTA and MRA rather than 3DRA.

Recently, computational fluid dynamics (CFD) plays an important role in researching cerebral aneurysm. Different imaging characteristics of CTA, MRA and 3DRA may have an impact on the reconstruction of the aneurysm models in the CFD simulation. Comparison studies of the model generation from different images have been presented in previous work [15, 16]. A previous study pointed out that even if the reconstructed aneurysm model has minor changes, there might still be a large difference in the hemodynamic results [17]. The purpose of this study is to evaluate the difference in both model geometry and fluid mechanics parameters between the reconstructed models from CTA, MRA and 3DRA.

## Methods

### Patients and imaging

Clinical study was performed at the Department of Radiology in Nanjing General Hospital. Ten patients (three males and seven females; mean age 56.4 years) with saccular cerebral aneurysms were enrolled in the study.

3DSA image data were obtained from Axiom Aritistd TA digital subtraction angiography (Siemens Medical Systems, Germany). The reconstruction of artery was performed in the 3D-image post processing workstation (Siemens). CTA image data were obtained from Somatom Definition AS 128 spiral CT scanner (Siemens Medical Systems, Germany). MRA image data were obtained from Achieva_1.5T Nova-Dual magnetic resonance (Philips Healthcare, The Netherlands), using maximum intensity projection (MIP) and multi-planner reformation (MPR) method to process vascular image reconstruction.

All patients with incidental findings of cerebral aneurysms at MRA and CTA underwent further evaluation of 3DRA within a 5-day interval. In this way we obtained the image data of 3DRA, CTA and MRA for each patient. Three of the ten aneurysms were found to be ruptured. The patient characteristics and aneurysm locations are shown in Table 1. This study was approved by the internal review board (Research Committee for Clinical Research of Zhongda Hospital, Affiliated to Southeast University), and informed written consent was obtained.

**Table 1:** Patient characteristics and aneurysm location

No	Patient				Aneurysm	
	Sex	Age	Smoking	Hypertension	Location	Rupture
1	F	60	No	No	LICA	No
2	F	59	No	Yes	LICA_PCoA	Yes
3	M	61	Yes	No	LICA	No
4	F	45	No	Yes	RICA	Yes
5	F	46	No	Yes	AcoA	Yes
6	M	49	No	No	RICA_PCoA	No
7	F	68	No	No	LACA_A1	No
8	M	75	No	No	LICA_PCoA	No
9	F	64	No	Yes	RPCoA	No
10	F	37	No	No	LICA_O_ph_A	No

### Model reconstruction

**Fig. 1:**
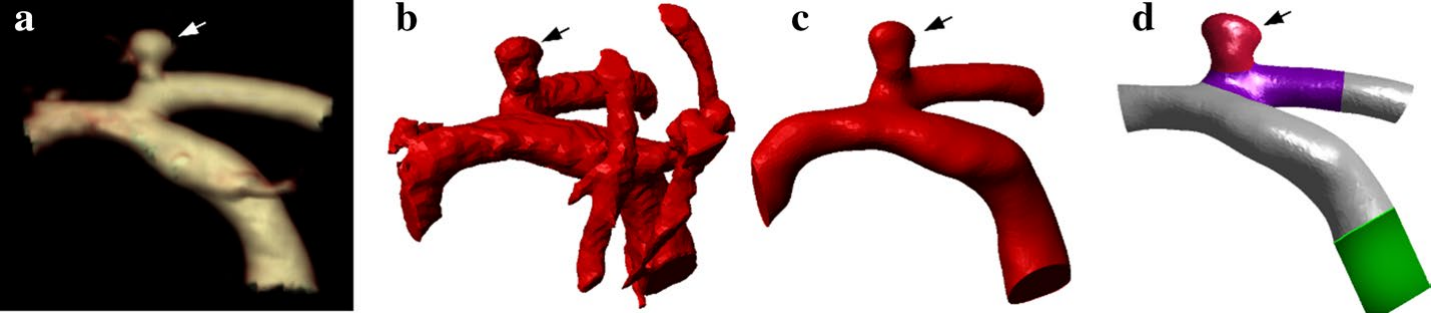
Model reconstruction of patient-specific model. **a** A original image data, **b** a rough model by using the threshold segmentation and artificially adjusting, **c** the final model after surface smoothing, **d** showing different areas in the model. *Arrows* represent the aneurysm

Three different patient-specific models were reconstructed for each patient based on the three different imaging modalities. In brief, 2D cross-sectional images of the cerebral aneurysms and its parent arteries were imported into ScanIP, Version 6.0 (Simpleware Ltd) for image segmentation and reconstruction. The segmentation procedure mainly detected the optimal boundary of the artery surface with different segmentation method. Firstly, a rough model was obtained by using the threshold segmentation, and then the algorithm of region-growing was used to remove the unconnected regions, conserving the aneurysm and its parent vessel. The small branches of the parent artery were removed artificially, based on the published studies [18]. Surface smoothing was performed with recursive Gaussian filter to reduce the sharp corners. Figure 1 shows the procedure of the patient-specific model development.

The vascular models were converted to a stereolithography (STL) format and exported to Workbench, Version 15.0 (ANSYS Inc.). In order to visualize and analyze the region of interests (ROI), for example, the aneurysm or the parent vessel, we divided the model into different regions. The ROI extent of the parent vessel applied to all models was 1.5 times of the diameter of the proximal vessel neck. In addition, an extended vessel was added to the inlet to allow the fully developed flow formation before entering the vascular model (Fig. 1d).

### CFD simulation

Unstructured meshes were created with ICEM CFD, Version 15.0 (ANSYS Inc.). The maximum element size was 0.25 mm with a minimum size of 0.1 mm for the high curvature regions and the surface of the aneurysms. On average, meshes of each model consisted of 0.15 million nodes and 0.85 million elements.

Blood was treated as incompressible, viscous Newtonian fluid (*ρ* = 1056 kg/m^3^, *μ* = 0.0035 Pa·s), and the vessel wall was assumed to be rigid with no-slip boundary condition. The range of Reynolds numbers at the inlets was 500–750. Transient CFD simulation was performed with Fluent, Version 15.0 (ANSYS Inc.), which uses a finite volume approach to solve the Navier-Stokes equations. Based on phase contrast (PC) MRA [19], a pulsatile volume flow waveform of a healthy volunteer in the ICA was imposed at the inlet. According to the inlet volume flow rate, a simple relation assuming constant radius was employed to obtain the mean blood velocity at the inlet section as:

1 $$v_{mean} \left( t \right) = \frac{q(t)}{{\pi (d/2)^{2} }}$$

where d is the inlet diameter, t is time and *v*_*mean*_ is the mean velocity. If the inlet was not located on the ICA, the waveform was scaled to simulate the physiologically realistic flow rate. The same inlet flow rate of the three models for each patient was used in the simulation. An outflow boundary with weight of 1 was used when the model had only one outlet. If there were two outlets, an appropriate flow rate relation was set based on the normal physiological blood flow distribution rate of different arteries. In this study, two outlets were the middle cerebral artery and anterior cerebral artery. The flow distribution ratio between MCA and ACA was 0.65:0.35 [20].

In the simulation, the convergence criterion was satisfied when the residual of continuity was less than 10^−4^ and the residual of velocity component was less than 5.0 × 10^−5^.

### Parameter comparison and analysis

Seven morphological parameters were investigated, namely size [21], aspect ratio (AR) [22, 23], neck area (NA), parent vessel diameter (PVD) [24], size ratio (SR) [24], aneurysm angle (AA) [24] and surface distance (Table 2).

**Table 2:** Definition of the morphological parameters

Morphological parameter	Abbreviation	Definition
Size	–	The maximum perpendicular height of the IA
Aspect ratio	AR	The ratio of the maximum perpendicular height to the average neck diameter
Neck area	NA	The minimum area of the neck
Parent vessel diameter	PVD	The diameter of the parent vessel
Size ratio	SR	The ratio of maximum aneurysm height to the PVD
Aneurysm angle	AA	The angle of inclination between the aneurysm and its neck plane
Surface distance	SD	The symmetrical Hausdroff distance between two models

**Fig. 2:**
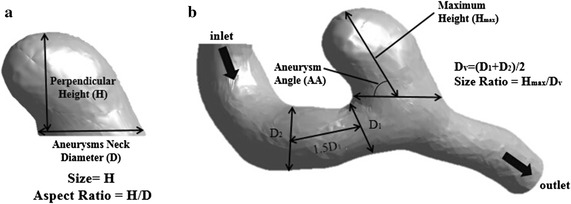
The illustration of the morphological parameters. **a** Definition of size and AR, **b** definition of AA and SR

When defining AR, the average neck diameter was calculated by the maximum diameter and the minimum diameter. Besides, the PVD was obtained by measuring two representative vessel cross sections of the parent vessel (D1 at the proximal neck and D2 at 1.5 × D1 upstream). The diameter of the parent vessel was calculated in the same way as the neck diameter. It must be emphasized that, when defining SR, the maximum aneurysm height was the distance from the centroid of the aneurysm neck to any point of the dome, rather than the maximum perpendicular height. The illustration of the morphological parameters and formulas are presented in Fig. 2. The surface distance, which was different compared with other six parameters, was calculated with a tool available in Amira, Version 5.4 (Visage Imaging Inc.). When calculating the surface distance, the two models must be approximately overlapped by using automatically aligned function. In this paper, 3DRA based models (DM) were treated as the “standard models”, CTA based models (CM) and MRA based models (MM) were compared with the “standard model” and the surface distances were calculated, respectively.

Nine hemodynamic parameters were studied, namely average wall shear stress (AWSS_A_, AWSS_P_), low wall shear stress (LWSS_A_), maximum wall shear stress (MWSS_A_), 90th percentile value of wall shear stress (90WSS_A_), oscillatory shear index (OSI_A_), maximum oscillatory shear index (MOSI_A_), high oscillatory shear index (HOSI_A_) and average wall shear stress gradient (AWSSG_A_). The subscript “A” and “P” represents aneurysm and parent vessel, respectively. These nine hemodynamic parameters were derived from three main parameters: AWSS, OSI and AWSSG. They were defined as:

2 $${\text{AWSS}} = \left| {\frac{1}{T}\int\nolimits_{0}^{T} {\overrightarrow {WSS} dt} } \right|$$

3 $${\text{OSI}} = \frac{1}{2}\left( {1 - \frac{{\left| {\frac{1}{T}\int_{0}^{T} {\overrightarrow {WSS} } } \right|dt}}{{\frac{1}{T}\int_{0}^{T} {\left| {\overrightarrow {WSS} } \right|dt} }}} \right)$$

4 $${\text{AWSSG}} = \frac{1}{T}\int\nolimits_{0}^{T} {\sqrt {\left| {grad(WSS_{x} )} \right|^{2} \,+\, \left| {grad(WSS_{y} )} \right|^{2} + \left| {grad(WSS_{z} )} \right|^{2} d}}$$

The LWSS_A_ was defined as the portion of aneurysm wall under low WSS (<0.4 Pa) at the end diastole [25] and the MWSS_A_ represented the maximum WSS on the aneurysm wall at the peak systole [26]. These two parameters were believed to be important in the rupture of the aneurysm. The 90WSSA calculated the 90th percentile value of the WSS on the aneurysm at peak systole [27]. OSI is a non-dimensional parameter with a value between 0–0.5, and it is the measurement of the directional change of WSS during the cardiac cycle [28]. MOSIA and HOSIA refer to the maximum OSI and the portion of aneurysm wall above the high OSI (>0.2) at the peak systole, respectively.

According to the classification proposed by Cebral et al, three flow characteristics were compared in this paper, naming flow pattern (FP), size of the impingement region (IS) and size of the inflow jet (JS), respectively. Basedon the streamlines of velocity and the distribution of WSS and OSI, two independent observers assessed the three flow characteristics and a third observer re-examined when the first two had different results.

Two observers independently performed the measurement of the morphological parameters of different patient-specific models and the mean values were used for analysis. The hemodynamic parameters were obtained by Fluent and Tecplot 360, Version 2014 (Tecplot Inc.). CFD-Post, Version 15.0 (ANSYS Inc.) was used for visualization and the assessment of flow characteristics.

3DRA was considered as the gold standard in detecting the intracranial aneurysms. Patients were imaged more than once with each imaging modality and that the models based on 3DRA showed the least variance. Therefore, in this study, we treated the DM as the “standard model” and other two models (CM and MM) were compared with DM, respectively. The difference of each parameter was calculated as: (|CM (or MM) − DM|)/DM × 100 %. The means and standard errors of all morphological and hemodynamic parameters were calculated. The differences between DM and CM or DM and MM were analyzed by a paired nonparametric Wilcoxon test. Differences were considered statistically signification for P ≤ 0.05. As for the assessment of flow characteristic, agreement between DM and CM or DM and MM were tested with Kappa test. Agreement was categorized as poor (k < 0.4), moderate (0.4 < k < 0.75), or good (k > 0.75). Statistical analyses were performed using SPSS 19.0.

## Results

### Comparison of morphological parameters

**Fig. 3:**
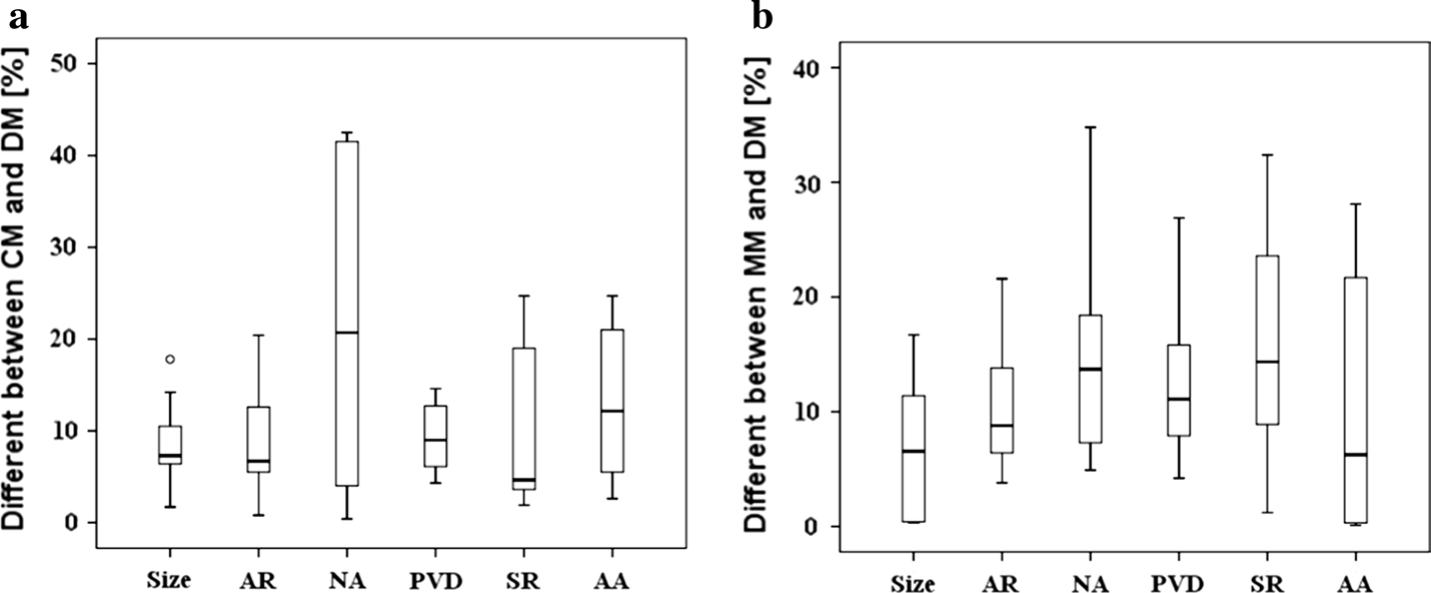
Differences distribution for morphologic parameters. **a** Difference between CM and MM, **b** difference between MM and DM

All values for means and SEs for each morphological parameter are displayed in Table 3. Figure 3a presents the boxplot about difference of the morphology parameters between CM and DM. Wherein, AR had the minimum difference (Δ = 8.3 ± 1.39 %, P = 0.953), followed by size (Δ = 8.7 ± 1.39 %, P = 0.333). However, NA had the maximum difference (Δ = 21.5 ± 5.5 %, P = 0.799) with the biggest difference over 40 %. Generally, CM was similar with DM in the above morphological parameters with the maximum difference less than 25 %, except for NA. It may be the partial volume effect in CT imaging which had the biggest influence of measuring the area. As a result, the NA may be overestimated in modeling.

**Table 3:** Differences between models of morphologic parameters (mean ± SE)

	Differences		P value	
	CM and DM (%)	MM and DM (%)	CM and DM	MM and DM
Size	8.7 ± 1.39	6.6 ± 1.85	0.333	0.683
AR	8.3 ± 1.72	10.2 ± 1.66	0.953	0.203
NA	21.5 ± 5.5	15.4 ± 2.74	0.799	0.169
PVD	9.38 ± 1.08	12.07 ± 1.99	0.114	0.575
SR	9.49 ± 2.54	15.48 ± 3.09	0.959	0.760
AA	13.27 ± 2.46	10.13 ± 3.18	0.333	0.646

Figure 3b shows the boxplot about difference between MM and DM. In these parameters, size had the minimum difference (Δ = 6.6 ± 1.85 %, P = 0.683). On the whole, the distribution range of differences was greater, suggesting that MRA had more disturbances in imaging the intracranial aneurysm. Nevertheless, the maximum difference was less than 30 % and the measuring results could also reflect the basic geometry of the aneurysm.

**Fig. 4:**
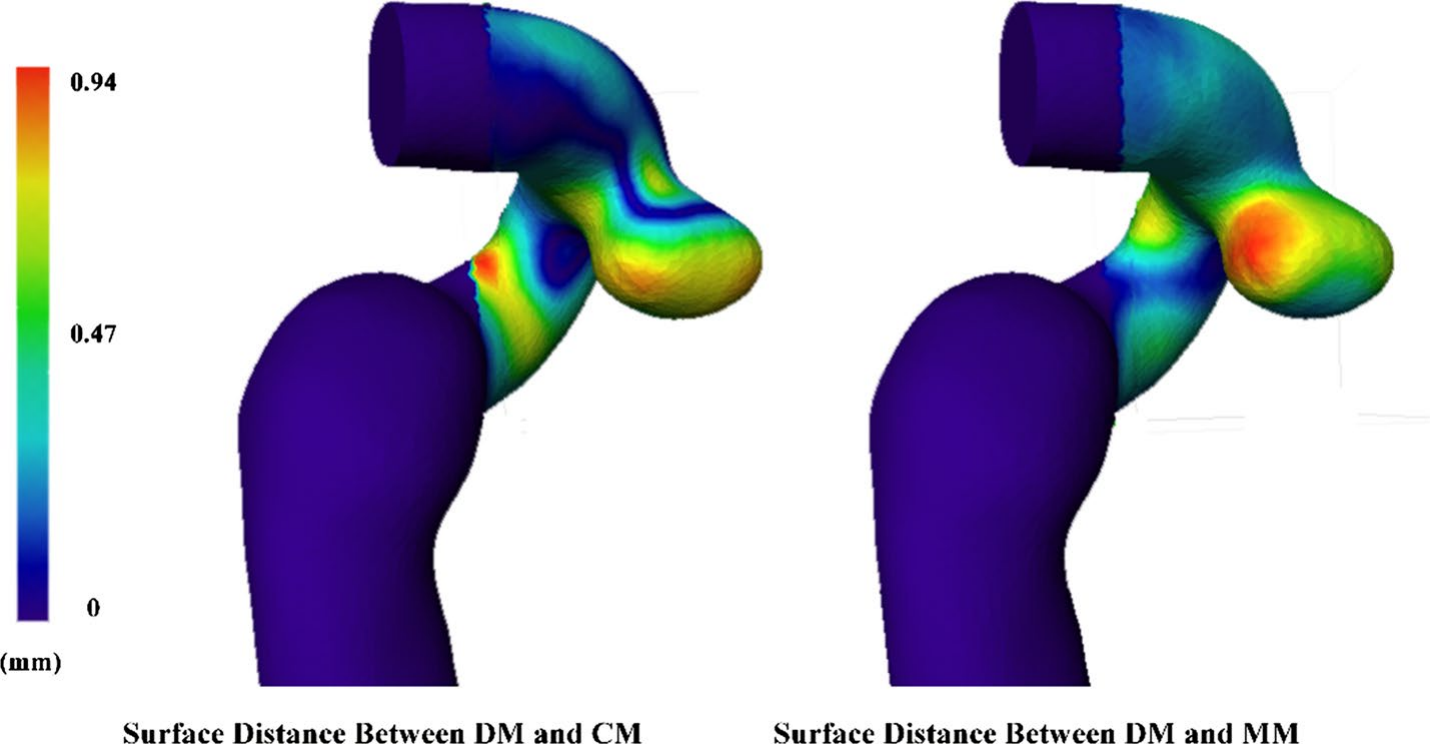
Surface distance distribution of a cerebral aneurysm located in ICA

**Fig. 5:**
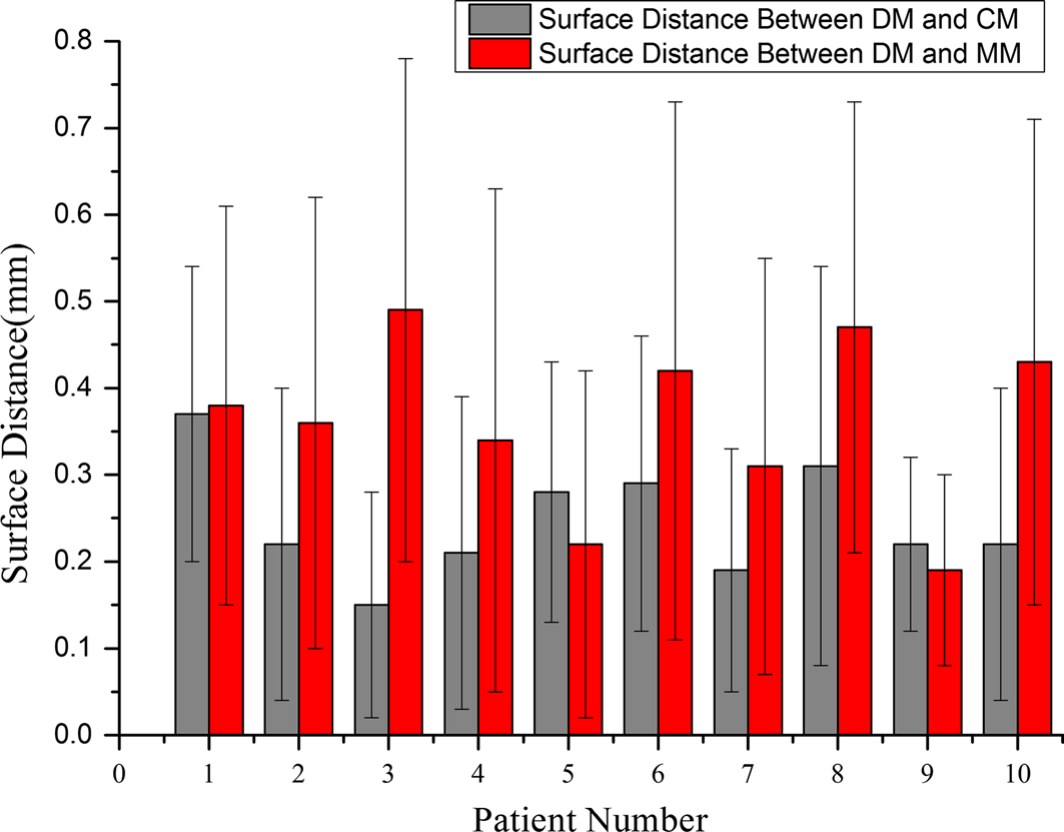
Surface distance data (mean and standard deviation) of the patients

Surface differences were presented in Fig. 4, which shows the distribution of the surface distance of a cerebral aneurysm located in ICA between CM (left), MM (right) and DM, respectively (patient No. 1). Figure 5 shows the surface distance (mean and standard deviation) of the ten patients. As for the surface distance between DM and CM (grey column), the maximum mean distance occurred in patient 1 (0.37 ± 0.17 mm) and the minimum mean distance occurred in patient 3 (0.15 ± 0.15 mm). As for the surface distance between DM and MM (red column), the maximum and the minimum mean distance occurred in patient 3 (0.49 ± 0.29 mm) and patient 9 (0.19 ± 0.14 mm). DM was more closely approximated by CM than by MM. The mean surface distance of the ten patients was (0.25 ± 0.07 mm) for CM and (0.36 ± 0.1 mm) for MM.

### Comparison of hemodynamic parameters

**Fig. 6:**
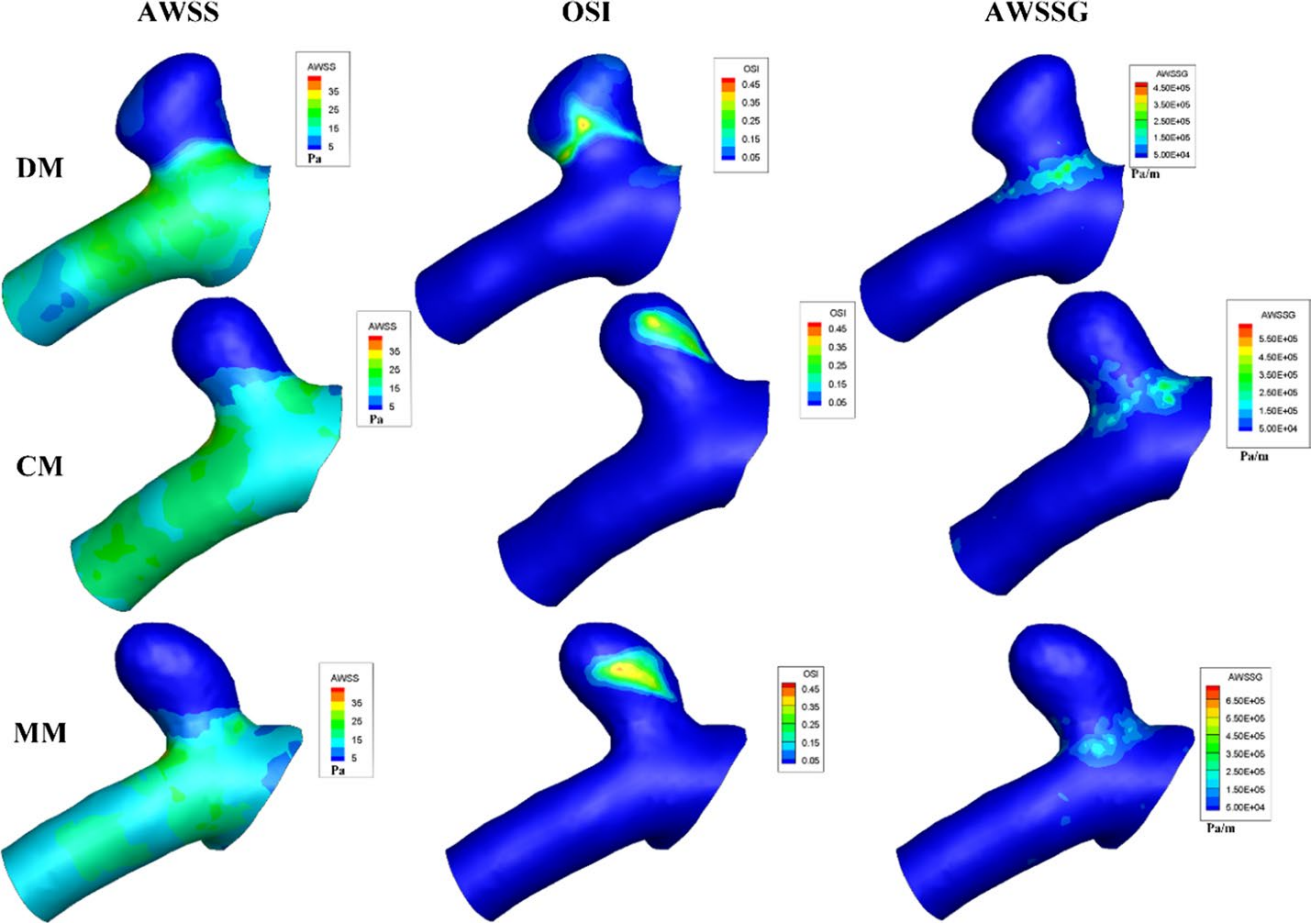
The distribution of AWSS, OSI and AWSSG in the three image based models (DM, CM and MM)

Figure 6 shows the AWSS, OSI and AWSSG maps of a cerebral aneurysm located in ACA-A1 and its parent artery (patient No. 5). We can see all of the three models (DM, CM and MM) shared a similar distribution of the three hemodynamic parameters: low AWSS at the sack, high OSI at the body and high AWSSG at the neck. Similar phenomena can be found in the other nine cases.

**Fig. 7:**
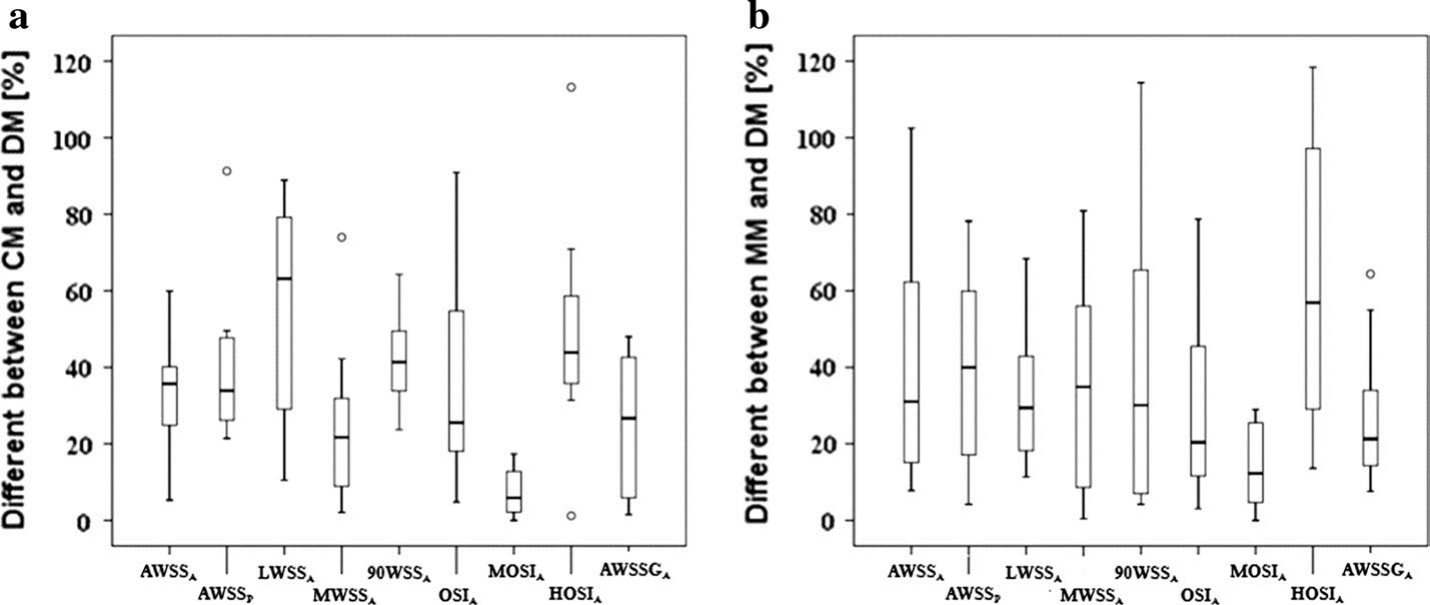
Differences distribution for hemodynamic parameters. **a** Difference between CM and DM, **b** difference between MM and DM

The differences information boxplot of nine hemodynamic parameters is presented in Fig. 7. All quantitative values for means and SEs for each hemodynamic parameter were displayed in Table 4. Overall, the differences of hemodynamic parameters appeared to be relatively significant, especially for the parameter of HOSI. AWSS_A_ was usually used when studying the hemodynamics of aneurysm. Here, there was a large variation in the average WSS, either the difference between CM and DM (Δ = 34 ± 5.13 %, P = 0.646) or between MM and DM (Δ = 40.6 ± 9.21 %, P = 0.575). Compared with other parameters, the difference of MOSI_A_ was smaller relatively (Δ = 7.3 ± 1.79 % for CM, Δ = 13.8 ± 3.13 % for MM). Meanwhile, in some cases, the difference about HOSI has the biggest value which is larger than 100 % in both two charts.

**Table 4:** Differences between models of hemodynamic parameters (mean ± SE)

	Differences		P value	
	CM and DM (%)	MM and DM (%)	CM and DM	MM and DM
AWSS_A_	34 ± 5.13	40.6 ± 9.21	0.646	0.575
AWSS_P_	40.3 ± 6.21	40.4 ± 7.68	0.169	0.508
LWSS_A_	53.4 ± 8.91	32.9 ± 5.89	0.285	0.575
MWSS_A_	25.3 ± 6.3	34.8 ± 8.56	0.575	0.878
90WSS_A_	42.6 ± 3.65	37.9 ± 10.67	0.445	0.959
OSI_A_	38.2 ± 8.6	29.9 ± 7.69	0.799	0.646
MOSI_A_	7.3 ± 1.79	13.8 ± 3.13	0.766	0.859
HOSI_A_	49.49 ± 8.76	63.35 ± 11.77	0.646	0.959
AWSSG_A_	24.8 ± 5.14	26.8 ± 5.73	0.093	0.386

### Comparison of flow characteristics

**Fig. 8:**
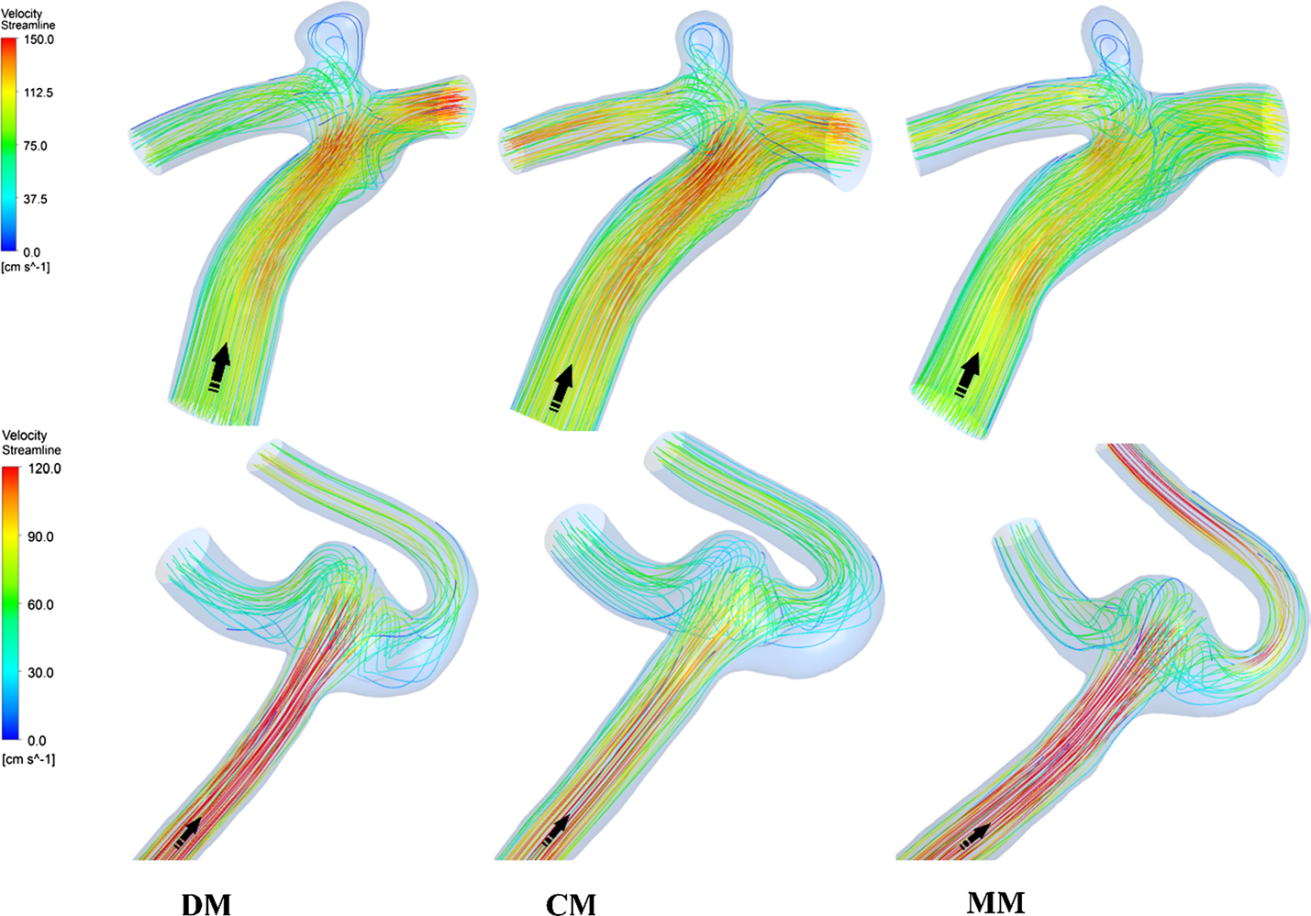
Velocity-colored streamline at the peak systole of two aneurysms located on ACA-A1 (*top row*) and ACoA (*bottom row*). Models based on different imaging techniques were present from *left* to *right* (DM, CM and MM).The *black arrow* shows the flow direction

Figure 8 shows the streamline colored with velocity magnitude at the peak systole of two aneurysms (patient No. 4, No. 5). As for the case located on ACA-A1 (top row in Fig. 8), they had the same FP (unchanging direction of inflow jet with single stable vortex), same IS (large) and same JS (large). As for the case located on ACoA (bottom row in Fig. 8), all the three models shared the same FP (unchanging direction of inflow jet with multiple stable vortices), but the IS and JS were small for CM while they were lager for DM and MM. Other aneurysms were also assessed in this way and the results are shown in Table 5. Both CM and MM had a good agreement in assessing the flow characteristics when comparing with DM.

**Table 5:** Agreement judgment of FP, IS and JS between different image based models

Parameter	No. of categories	Agreement (k)		
		DM and CM	DM and MM	CM and MM
FP	4	1	0.79	0.79
IS	4	0.78	1	0.78
JS	2	0.78	0.78	0.58

## Discussion and conclusion

As for intracranial aneurysm, both morphological and hemodynamic parameters are closely associated with the process of aneurysm formation, progression and rupture. Each imaging modality has its own unique features which may affect the measurement of morphological parameter and consequently hemodynamic parameters [29, 30]. 3DRA has greater patient risk and discomfort because it is more invasive than the others. Moreover, 3DRA is highly sensitive to the inhomogeneity caused by the contrast agent filling process during acquisition [31]. In CTA images, it may be hard to segment near-bone vasculature because the contrast agent and the intensity values overlap which consequently affects the accuracy of model reconstruction from CTA images. In MRA images, there is some signal loss intra large aneurysms with disturbed or slow flow and therefore cannot display the entire structure of the aneurysm [32]. Although 3DRA is the gold standard, CTA and MRA are still widely used in the diagnosis and treatment of aneurysms in clinical. In general, CTA is usually used in diagnosis and follow-up studies, and 3DRA is usually used before and during treatment [33]. MRA is also used because of its advantages such as cheap, non-invasive and non- radioactive. In our study, 3DRA was used as the reference standard because it has the highest spatial resolution and lowest visibility [14, 34, 35] of bone which result in better anatomic accuracy among the three modalities [12, 36].

Comparison studies of the model generation from different images have been presented in previous work. Goubergrits et al. have compared the difference of 3DRA, CTA and MRA in cerebral aneurysm geometry and hemodynamics based on an in-vitro study. They found that all three imaging modalities adequately reproduce aneurysm geometry and allow meaningful CFD analyses [12]. Piotin et al. [26] have assessed the impact of CTA, MRA and 3DRA for measuring the volume of an in-vitro aneurysm model and found that 3DRA is more accurate than CTA, while CTA is more accurate than MRA. However, most of the studies above were based on in-vitro experiment and had a phantom model to be compared. Based on ten aneurysms imaged with 3DRA and CTA, Geers et al. [20] have evaluated the impact of imaging technique on model reconstruction and CFD simulation. Geers’ results show that, although differences of quantitative hemodynamic variables were relatively large, the main flow characteristics were in excellent agreement. This is consistent with our findings. Meanwhile, in the recent research about hemodynamics impact related to differences in geometry, Schneider et al. [15] evaluated the neck size overestimation’ effect on hemodynamic features. They found that the lager neck size can result in a different impingement region and the area of low WSS. The study of Hoiet al. [16] also showed that differences in neck size can result in different flow patterns. In this paper, we find patient No. 3 has the largest surface distance difference, meanwhile its hemodynamic parameters (like LWSS_A_, OSI_A_, HOSI) have similar largest differences. This is suggest that differences in geometry are related to hemodynamics results.

The purpose of this study is to evaluate the difference in model geometry and fluid mechanics parameters of reconstructed models from 3DRA, CTA, and MRA. To our knowledge, this is the first in-vivo study to assess the impact of the three imaging modalities on model reconstruction and CFD simulation. Ten aneurysms, imaged with 3DRA, CTA and MRA, were reconstructed to computational model DM, CM and MM, respectively. Seven morphologic parameters and nine hemodynamic parameters were chosen to compare and analyze the effects of different imaging method on the modeling and CFD simulation.

In the comparison of morphology, CM has a better reproducibility than MM for most parameters. Difference between CM and DM mainly lies within 15 % with biggest difference of nearly 25 %, except for NA. Although MM has a bigger difference range, it has also an acceptable reproducibility with biggest difference less than 35 %. These differences may be resulted from different imaging technique or artificial measurement errors. However, all of the three modalities has no significant differences and allow meaningful measurement of morphologic parameters. In the comparison of hemodynamics, DM, CM, MM share a similar distribution of the main hemodynamic parameters, low AWSS at the sack, high OSI at the body and high AWSSG at the neck. However, quantitative comparison reveals that both CM and MM have significant difference from DM. Especially, the difference about HOSI has the biggest value which is larger than 100 % in some cases. This is because even though there are only small differences in morphology of some individuals, those small differences can result in obvious differences in hemodynamic parameters.

In conclusion, 3DRA, CTA and MRA have no significant differences in reproducing intracranial aneurysm geometry. The CFD results suggests there might be some significant differences in hemodynamic parameters between the three imaging-based models and this needs to be considered when interpreting the CFD results of different imaging-based models. If we only need to study the main intra-aneurysmal flow patterns, three types of image-based model might be all suitable for patient-specific computational modeling studies.
